# Lipidomic and Spatio-Temporal Imaging of Fat by Mass Spectrometry in Mice Duodenum during Lipid Digestion

**DOI:** 10.1371/journal.pone.0058224

**Published:** 2013-04-03

**Authors:** Alexandre Seyer, Michela Cantiello, Justine Bertrand-Michel, Véronique Roques, Michel Nauze, Valérie Bézirard, Xavier Collet, David Touboul, Alain Brunelle, Christine Coméra

**Affiliations:** 1 Centre de Recherche de Gif, Institut de Chimie des Substances Naturelles, Centre National de la Recherche Scientifique (CNRS), Gif-sur-Yvette, France; 2 Institut National de la Santé et de la Recherche Médicale (INSERM) U563, Hôpital Purpan, Toulouse, France; 3 Institut National de la Santé et de la Recherche Médicale U1048, Hôpital Rangueil, Toulouse, France; 4 Institut National de la Recherche Agronomique (INRA) UMR 1331, TOXALIM, Toulouse, France; National Institute of Agronomic Research, France

## Abstract

Intestinal absorption of dietary fat is a complex process mediated by enterocytes leading to lipid assembly and secretion of circulating lipoproteins as chylomicrons, vLDL and intestinal HDL (iHDL). Understanding lipid digestion is of importance knowing the correlation between excessive fat absorption and atherosclerosis. By using time-of-flight secondary ion mass spectrometry (TOF-SIMS), we illustrated a spatio-temporal localization of fat in mice duodenum, at different times of digestion after a lipid gavage, for the first time. Fatty acids progressively increased in enterocytes as well as taurocholic acid, secreted by bile and engaged in the entero-hepatic re-absorption cycle. Cytosolic lipid droplets (CLD) from enterocytes were originally purified separating chylomicron-like, intermediate droplets and smaller HDL-like. A lipidomic quantification revealed their contents in triglycerides, free and esterified cholesterol, phosphatidylcholine, sphingomyelin and ceramides but also in free fatty acids, mono- and di-acylglycerols. An acyl-transferase activity was identified and the enzyme monoacylglycerol acyl transferase 2 (MGAT2) was immunodetected in all CLD. The largest droplets was also shown to contain the microsomal triglyceride transfer protein (MTTP), the acyl-coenzyme A-cholesterol acyltransferases (ACAT) 1 and 2, hormone sensitive lipase (HSL) and adipose triglyceride lipase (ATGL). This highlights the fact that during the digestion of fats, enterocyte CLD contain some enzymes involved in the different stages of the metabolism of diet fatty acids and cholesterol, in anticipation of the crucial work of endoplasmic reticulum in the process. The data further underlines the dual role of chylomicrons and iHDL in fat digestion which should help to efficiently complement lipid-lowering therapy.

## Introduction

Prolonged and exaggerated postprandial plasma triacylglycerol (TAG) concentrations and hypercholesterolemia are risk factors for cardiovascular disease or metabolic syndrome [1], [2], [3]. This is clearly related to changes in dietary habits leading to overconsumption of lipids, associated to low physical activity. Absorption by the small intestine has a key role in this metabolism being the exclusive site of absorption of dietary and biliary cholesterol. It also determines the definitive cholesterol disposal in feces. The intestine absorbs 95% of ingested fat, which are mainly triglycerides (TAG), and 50% cholesterol being positively correlated with hypercholesterolemia [3], [4]. Consequently, in order to control excessive absorption, a better understanding of fat digestion process remains an important issue in human health.

Lipid uptake by intestinal enterocytes is a complex and regulated mechanism, which hosts three main stages [2], [4], [5]. At first, intestinal absorption involves the luminal lipolysis of triglycerides in 2-monoacylglycerols (2-MAG) and fatty acids (FA) and of esterified (EC) in free cholesterol (FC) and FA. Cholesterol, MAG and FA are then emulsified into mixed micelles by bile salts and pass through an unstirred water layer covering the enterocyte surface. In a second step, lipids are absorbed by the apical brush border membrane (BBM), through a partially understood mechanism which has strong evidence to be protein-mediated. The scavenger receptors CD36 and SR-BI contributes to the apical uptake of FA or FC [4], [5], [6], [7], [8], [9] whereas NPC1L1 (Niemann-Pick C1-Like 1 protein) is crucial for the absorption of cholesterol but not FA [9], [10], [11]. After absorption, lipids are addressed from the plasma membrane to the endoplasmic reticulum (ER), through a mechanism which involves FA-binding proteins (FABP) but certainly also NPC1L1 [12], [13], [14]. In a third step, the absorbed lipids are metabolized in the ER where FA, MAG and FC are esterified in TAG and EC [15] and transferred to apolipoprotein B48 by microsomal triglyceride transfer protein (MTTP) [15], [16]. This produces chylomicrons, (or vLDL) secreted to the lymph and which transport most of diet FA, and about 70% of cholesterol. The remaining 30% is also esterified, and secreted in circulation, as intestinal high density lipoproteins (iHDL) by a mechanism dependent on ABCA1 (ATB-binding cassette A1 transporter), but not MTTP [4], [5], [17].

During the postprandial period in enterocytes, the TAG are also stored temporarily in the cytosolic lipid droplets (CLD), which are believed to be also synthesized by the ER where most of lipid metabolism occur. CLD are well known in adipocytes but were also recently identified in many other cell types. They are composed of a fatty core rich in TAG and EC, surrounded by a surface layer of phospholipids, cholesterol and proteins. With a similar composition, plasma lipoproteins are actually secreted or extracellular LD with the peculiarity of being surrounded by apolipoproteins. CLD and lipoproteins have various densities and sizes of several nanometers to several thousand, depending in TAG contents. Intestinal CLD where found to store the absorbed lipids notably as TAG but also either adipophilin or TIP-47 [18], [19]. The proteomic or lipidomic characterization of CLD revealed that they actively participate to the exchange of lipids and proteins, between different organelle membranes, and participate to lipid metabolism [20], [21], [22], [23]. This knowledge must still be completed to understand similarities and differences in the structures and functions of CLD between various cells or tissues and for our purpose, those produced in enterocytes during fat digestion.

We first characterized the traffic of fat, at different times of the digestion in mouse intestine, using time-of-flight secondary ion mass spectrometry imaging (TOF-SIMS). Among all mass spectrometry imaging techniques (MALDI-TOF, nano-SIMS, DESI...), this method allows a concomitant localization of several intact molecules (up to 1000–1500 Da), and especially lipid species of animals or human samples, with a lateral resolution ranging from several microns to less than 400 nm [24], [25], [26], [27], [28], [29], [30], [31].

Intestinal LD were visualized by histology, isolated and then, chemically characterized for their lipid contents. Our results show that they span a wide range of sizes and densities and provide a dynamic storage of absorbed lipids. Interestingly and unlike the majority of CLD, they contain fatty acids, not only as completely metabolized in TAG, but also as intermediate compounds such as DAG, MAG and FFA. They therefore appear as original transient lipid structures allowing to accumulate and transform the absorbed lipids into the enterocyte cytosol, upstream the synthesis of chylomicrons and HDL.

## Materials and Methods

### Mouse Intestinal Absorption of Lipids

All animal studies were performed in conformity with the public Health Service Policy on Human Care and Use of Laboratory Animals and in accordance to the local ethics committee (Inserm/CPTP, CHU Purpan, Toulouse) and with its approval. C57BL/6 Rj mice were purchased from Janvier (Janvier, Le-Genest-St-Isle, France) and housed in a temperature-, humidity- and light-controlled room. They were given a standard chow diet with water ad libitum. To study digestion, 8–15-week-old mice were fasted overnight and forced-fed with 200 µL of 2% (p/v) cholesterol (Sigma) in sunflower oil or 200 µL of H_2_O for controls (T0 for no lipid digestion). Animals were sacrificed after 30 min (T0.5), 1 (T1) or 4 hours (T4) of digestion after gavage. For mass spectrometry analysis, the duodenum was removed, quickly rinsed with phosphate-buffered saline (PBS) and frozen in liquid nitrogen then −80°C. For other analyses, the duodenum and proximal jejunum (until 10 cm after the bile duct) was recovered and more intensely washed with 5 mM taurocholic acid (TC) in PBS to remove external lipids. It was directly frozen in isopentane for histological studies. Otherwise, it was longitudinally opened and the mucosa was gently scraped, immediately frozen and stored at −80°C.

### Mass spectrometry imaging

Twelve micrometers thick sections (from one mouse per time point) were prepared at −20°C with a CM3050-S cryostat (Leica Microsystems SA, Nanterre, France) and deposited onto silicon wafers (2°in. diameter polished silicon wafers; ACM, Villiers-Saint-Frédéric, France). Before analysis, samples were dried under vacuum at a pressure of a few hectopascals for 10 min. Optical images were recorded with an Olympus BX51 microscope fitted with ×1.25 to ×50 lenses (Olympus, Rungis, France) and a ColorView I camera monitored by Cell^B^ software (Soft Imaging System, GmbH, Münster, Germany).

All ion images were recorded using a TOF-SIMS IV commercial mass spectrometer (Ion-Tof GmbH, Münster, Germany). This spectrometer is equipped with a liquid metal ion gun delivering bismuth clusters. Bi_3_^+^ ions were selected for these experiments as they provide the best compromise between intensity and efficiency [32]. After being extracted from the source emitter with a potential of 25 kV, primary ions reach the sample surface at an angle of incidence of 45°. Secondary ions are accelerated to an energy of 2 keV, fly through a field free region and are reflected (effective flight path ∼2°m) before being post-accelerated to 10 keV just before hitting the surface of the hybrid detector, which is composed of one micro-channel plate, a scintillator and a photomultiplier. A low energy electron flood gun is activated between two primary ions pulses in order to neutralize the sample surface with the minimum damage [33]. Two modes of operation of the primary ion source have been used. The first one, called ‘high-current bunched mode’, allowed both a beam focus of 2 µm and a pulse duration of less than 1 ns, ensuring a good mass resolution (about M/ΔM  = 5×10^3^ FWHM at *m/z* 500). In the second mode of operation, called ‘burst alignment’, the primary ion beam is not bunched, so the pulse duration reaches 20–100 ns leading to a nominal mass resolution, but it can be focused to ∼400 nm, ensuring a high spatial resolution. The Bi_3_^+^ ion current measured at the surface of a Faraday cup located on the grounded sample holder was 0.5°pA in the high-current bunched mode and 0.05 pA in the burst alignment mode. The detailed description of these two modes of operations can be found elsewhere [34]. During the measurement, the primary ion beam is rastered over the sample surface, and a full TOF mass spectrum is recorded for each pixel. For all acquisitions, the number of pixels was 256×256.

Images in positive and negative ion modes with a field of view of 500×500 µm^2^ and with a lateral resolution of 2 µm were recorded in the high-current bunched mode. The primary ion dose density (also called ‘fluence’) was 5×10^11^ ions.cm^−2^, well below the static SIMS limit [35], ensuring little damage and high secondary ion emission for the acquisition of images of both polarities on the same area. Images of 100×100 µm^2^ with a lateral resolution of 390 nm were recorded in the negative ion mode using the burst alignment mode. The experiment was performed twice on separate tissue areas. Under these conditions, the fluence was 2×10^12^ ions.cm^−2^. The data acquisition and processing software were IonSpec and IonImage (TOF-SIMS 4.1, Ion-Tof GmbH, Münster, Germany). Ion peak assignments were made according to mass spectra of reference compounds, and confirmed by comparison with literature [23], [24], [27], [28], [29], [30].

### Histological staining of lipid droplets

The neutral lipids of intestinal LD were stained using Oil red O as previously described [36]. Transversal frozen tissue slices of 5 µm were obtained by cryosectioning at −25°C, adhered onto glass slides, air dried for 30 min, fixed for 10 min in 3.7% formol and rinsed twice in PBS. A staining solution was freshly prepared by adding 4 ml H_2_O in 6 mL of 0.2% oil red O (ORO, Sigma) in pure isopropanol Tissue slices were rapidly immersed in 60% isopropanol and in staining solution, for 10 minutes. They were then rinsed in 60% isopropanol, counterstained using Mayer's haematoxylin for 10 sec, mounted in 10% glycerol in PBS and captured by optical microscopy. Only lipids inserted into the tissue were visible while others were washed out during the process.

### Intestinal lipid droplet isolation

The intestinal mucosa (12 first proximal cm) of eight mice were harvested after 4 h of lipid digestion, when the intestine were highly enriched in enterocytic CLD and chylomicrons. Tissues were homogenized in ice by using an Ultra Thurrax blender in 0.8 mL of PBS supplemented with 5 mM EDTA, 20 µM of the lipase inhibitor orlistat (Sigma) and protease inhibitors: AEBSF 6 mM, aprotinin 2.4 µM, leupeptin 80 µM, bestatin 160 µM, pepstatine A 70 µM and E-64 28 µM and centrifuged at 4°C, 250 g for 10 min to eliminate tissue debris. The remaining supernatant was named I for intestinal homogenate and was used to purify three populations of lipid droplets, according to their respective sizes and densities, using sequential ultracentifugations. Each LD fraction was isolated by flotation on the surface of a top cushion of PBS at density d = 1 or 1.21. The method is based on the common protocol used to isolate plasma lipoproteins chylomicrons and HDL at 4°C, with the ultracentrifuge TL100 (Beckman) and rotor TL100-4 [37], [38]. The homogenization released cytosolic and secreted LD whereas the chylomicrons present in the secretory apparatus of enterocytes remained confined in microsomes. The first centrifugation at 20,000 g for 30 min. provided large LD, named D1, having similar size and density as chylomicrons. After a second ultracentrifugation at 100,000 g for 1 h, smaller LD named D2 were recovered on top cushion. At this step, the pellet was also recovered in a additional fraction named M, which contained intestinal membranes and microsomes. The remaining infranatant was equilibrated with KBr to a density of 1.21, overlaid by a cushion of phosphate buffer at d = 1.21 and subjected to a last ultra-centrifugation for 14 h at 120,000 g and 4°C. The top fraction D3 were harvested and contained droplets of small sizes and of densities between 1 and 1.21, including HDL-like structures (1.063<D<1.21). In some experiments the final infranatant S containing cytosolic components was also collected. Protein concentration was quantified using the Biorad protein assay.

#### Lipid quantification

Lipids corresponding to I, D1, D2, D3, M and S fractions were extracted according to Bligh and Dyer [39] in chloroform/methanol/water (2.5/2.5/2.1, v/v/v). Chloroform phases were evaporated to dryness. Molecular species of neutral lipids (FC, EC, DAG, TAG) were quantified by gas liquid chromatography [40]. Other phospholipids, i.e. phosphatidylcholine (PC) and sphingomyelin lipid extracts were analyzed by HPLC (DIONEX Summit) on a Uptisphere6OH analytical column (5 µm particle size, 250×2.1 mm) fitted with a DIOL guard column cartridge (10×2.1 mm; INTERCHIM) and coupled to a light scattering detector (Polymer Laboratory ELS 2100, nitrogen flow 1.8 mL/min, evaporating temperature 50°C, and nebulizer temperature 80°C). Separation was achieved at a flow rate of 0.25 mL/min using a gradient from 5 to 35% of B. (isopropanol/water/triethylamin:acetic acid (85/15/0.014/0.5, v/v/v/v)) in A (hexane/isopropanol/triethylamin:acetic acid (82/18/0.014/0.5, v/v/v/v) during 35 min. Neutral lipids has also been analyzed using thin layer chromatography (TLC) in 20/20 silica plates using hexane/diethylether/acetic acid (70/30/1, v/v/v) as the solvent. Lipids were stained with either iodine vapor or 25% H_2_SO_4_ associated to heating with an air dryer. They were identified by co-migrating lipid standards.

MGAT/DGAT activity assays. Assays were performed to follow the enzymatic reaction of MAGT or DAGT as described in [41]. Briefly, equivalent samples volumes of I (diluted 1/10), D1, D2, D3 and M (1/10) were adjusted to 10 mM Tris-HCl, pH 7.4, supplemented with 2 mg/mL of free fatty acid BSA, 50 µM of monooleylglycerol, 50 µM of diacylglycerol, 20 µM of oleoylCo A and 0.5 µCi of [14C]-oleoyl-CoA (Amersham, Les Ulis, France) and were incubated for 1 h at 37°C. The reaction was stopped with 5 mL of 1/1 chloroform/methanol, lipids were extracted and subjected to TLC as described above. After iodine vapor detection, the spots corresponding to FFA, MAG, DAG and TAG were scrapped off the plates and their radioactivity was quantified as for their synthesis by liquid scintillation counting.

### Western-blot

The proteins of isolated LD were precipitated with 10% final TCA on ice and centrifuged for 10 min at 20,000 g at 4°C, equilibrated in denaturating buffer (50 mM Tris/HCl, pH 6.8, 2% SDS (w/v), 15% glycerol (v/v), 2% β-mercaptoethanol (v/v), 2 M Urea and 0.02% (w/v) bromophenol blue) and heated for 10 min at 60°C. They were then separated by SDS-PAGE and electrically transferred onto nitrocellular membranes. Western blots were performed with anti-DGAT1 antibodies (Novus NB100-57086, 1∶1000), anti-MGAT2 (H-25, Santa-cruz, 1∶1000), anti-MTTP (N-17, Santa-cruz, 1∶200), anti-ACAT1 (Pierce A5 19227, 1∶500), anti-ACAT 2 (Origene TA501222, 1∶2000). For all immunoblots, the antibody binding was subjected to appropriate HRP-conjugated secondary antibodies, detected with ECL plus (GE Healthcare), and exposed to X-ray film (Hyperfilm ECL; GE Healthcare).

### Enzymatic activities

In order to detect possible contaminations by membranes, the LD were subjected to enzyme assays, identifying specific markers of enterocyte BBM (alkaline phosphatase and aminopeptidase N), the ER (NADPH cytochrome c reductase) and the Golgi apparatus (NADH cytochrome c reductase) using a spectrophotometer Varioskan (Fisher Scientific, Illkirch, France). All samples (I, D1, D2, D3, M, S) were equilibrated in 0.05% final of sodium deoxycholate (p/v) as lipid solubilizing detergent. Alkaline phosphatase activity was assayed as described earlier [42] in phosphate buffered saline (PBS) supplemented with 1 mM MgCl_2_ and using p-nitrophenyl phosphate as the substrate. The activity of aminopeptidase N was detected in 0.1 M Tris/HCl (pH 7.4) 1 mM CaCl_2_ by monitoring the increase in optical density at 405 nm of a solution containing 2.5 mM of alanine-p-nitroanilide (Ala-PNA, St Louis, MO) [43]. The assay system for reductase activities contained 0.05 M Tris/HCl, pH 7.5; 0.1 mM NADH or NADPH; 0.05 mM cytochrome c and 0.33 mM KCN. The reduction of cytochrome c was followed at 550 nm [44]. Enzyme activities (nmoles of substrate converted/mg of protein/min) were expressed as a percentage of the initial activity in the homogenate I.

## Results

### Lipid droplet visualization

**Figure 1:**
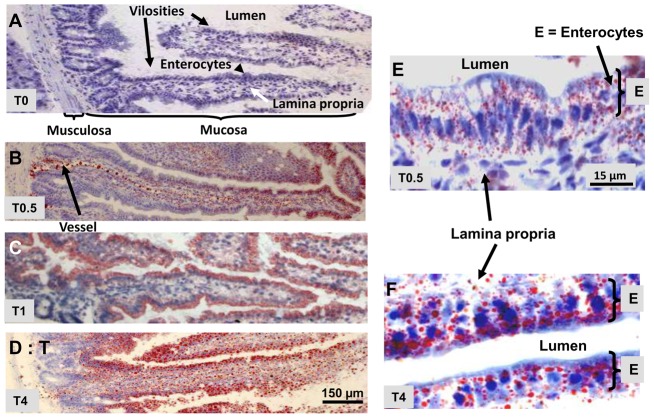
Neutral lipid staining by Oil red O of LD and chylomicrons in the duodenum of mice. Neutral lipids of frozen tissue slices, recovered at T0 (A), T0.5 (B, E), T1 (C) and T4 (D, F) of digestion after lipid gavage, were stained in red by ORO showing intestinal lipid droplets highly enriched in enterocytes, during digestion and chylomicrons, in the lamina propria. Membranes and intestine of starved mouse T0 were not stained.

At different times of digestion, the formation of lipid droplets in duodenum was monitored by optical microscopy, after staining of neutral lipids by ORO (Figure 1). This allowed the detection of large LD or chylomicrons having diameters of several hundred of nanometers. In starved animals (T0), no staining was observed in intestinal villi. At T0.5 after gavage, numerous LD stained in red were apparent, being mainly concentrated in the upper villus enterocytes, where absorption is predominant. The chylomicrons, secreted in mucosa or migrating along a lymphatic vessel were also visible. At T1 and T4, all enterocytes from the basement to the top of the villi, are highly stained, reflecting the abundance of LD or lipoproteins whereas secreted chylomicrons were apparent in the lamina propria.

### Mass spectrometry imaging

**Figure 2:**
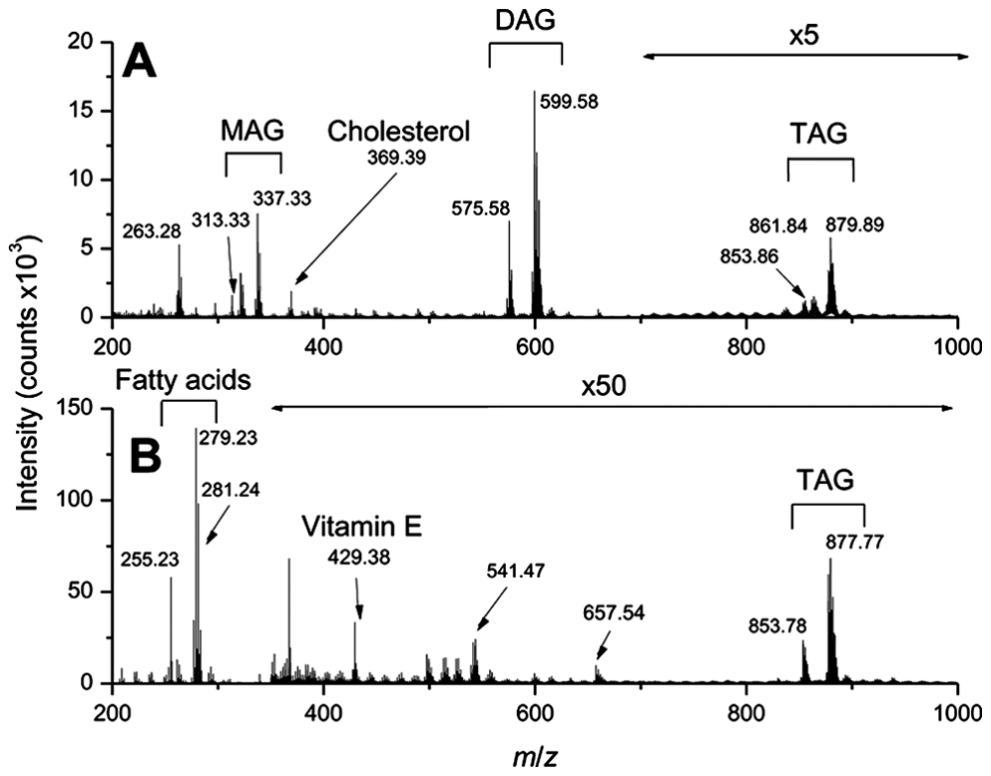
TOF-SIMS mass spectrum of the sunflower oil enriched in cholesterol (2% p/p). Spectra were recorded in positive (A) and negative ion modes (B).

Sunflower oil containing 2% of cholesterol (p/v) was first analyzed by TOF-SIMS in order to identify its various components. The oil was diluted by one to twenty in chloroform and deposited on a silicon wafer. TOF-SIMS mass spectra of the oil were recorded in the positive and negative ion modes (Fig. 2). It is known that the sunflower oil is mainly composed of triglycerides, containing about 60% of C18∶2 fatty acids, 22% of C18∶1 FA, 5% of C18∶0 and 6% C16∶0 FA [45]. They were detected in the positive ion mode mass spectrum (Fig. 2A), as ions of monoacylglycerols (MAG) containing C16∶0 and C18∶2 fatty acids (*m/z* 313.33 and *m/z* 337.33), diacylglycerols (DAG) bearing 34 (C16+ C18 FA) and 36 (C18+ C18 FA) carbon atoms on the two fatty acid chains (centered at *m/z* 575.58 and *m/z* 599.58, respectively) and triacylglycerol (TAG) ions containing either 50 carbon atoms on the three fatty acid chains (C16+ C16+ C18 FA, centered at *m/z* 853.86) and TAG bearing 52 carbon atoms (C16+ C18+ C18 FA, centered at *m/z* 861.84 and *m/z* 879.89). The peak at *m/z* 369.39 was attributed to cholesterol fragment ion [M+H-H_2_O]^+^. In the negative ion mode (Fig. 2B), signals at *m/z* 255.23, 279.23, 281.24 and 283.22 were the most intense and correspond to carboxylate ions of C16∶0, C18∶2, C18∶1 and C18∶0 fatty acids, respectively. Vitamin E (α-tocopherol; *m/z* 429.38) was also detected as well as TAG ion species containing 52 and 54 carbon atoms at *m/z* 853.78 and 877.77, respectively.

**Figure 3:**
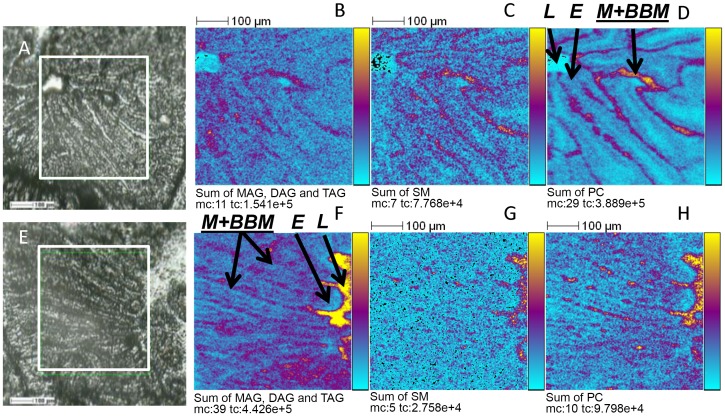
TOF-SIMS ion images recorded in positive ion mode over proximal intestine. Mice Tissues were recovered from mice at T0 (A to D) or T4 of digestion (F to H). A and E: optical pictures, ion images were recorded in the square delimited in white. B and F: sum of signals corresponding to monoacylglycerols (*m/z* 311.27, 313.29, 337.30, 339.31 and 341.34), diacylglycerol (*m/z* 573.54, 575.57, 577.59, 599.57, 601.59 and 603.53) and triacylglycerol (*m/z* 851.82, 853.82, 855.83, 877.72, 879.83, 881.84 and 883.85). C and G: sum of signals corresponding to sphingomyelin SM 34:1 (*m/z* 703.62, 725.64 and 741.59). D to H: sum of signals corresponding to phosphatidylcholines (*m/z* 734.66, 756.60, 782.61, 758.65, 780.62, 796.61, 786.66, 808.67, 824.64, 804.62 and 820.54). Primary ions Bi_3_^+^, 25 keV, fluence 5×10^11^ ions.cm^−2^, area of 500×500 µm^2^, 256×256 pixels, pixel size 2×2 µm^2^. The amplitude of the color scale corresponds to the maximum number of counts (mc) and could be read as [0, mc]. tc is the total number of counts recorded for the specified *m/z* (it is the sum of counts in all the pixels). L, duodenal lumen; M+BBM, Mucus + brush border membrane; E, enterocytes.

Duodenal sections recovered at different times of digestion, were then subjected to TOF-SIMS imaging to identify and localize endogenous and diet lipids. The major lipids detected in the positive ion mode corresponded to several mono-, di- and tri-acylglycerols, cholesterol, vitamin E, and some members of the sphingomyelin and phosphatidylcholine families. Signals corresponding to MAG 16 and MAG 18, DAG 34 and DAG 36 and TAG 52 and TAG 54 (see details in table 1), were summed in Figures 3B and 3F, since they shared similar localizations and are together representative of the fate. At T0, T0.5 and T1 (data for T0.5 and T1 not shown), they were located at the surface of intestinal villi corresponding to the luminal mucus and the adjacent brush-border membranes (BBM) of enterocytes, while at T4, they were very abundant in the lumen but also present in enterocytes Because of similar distribution profiles we also summed the images corresponding to the sphingomyelin family, containing 34 carbon atoms and one insaturation (*m/z* 703.62, [SM 34∶1+H]^+^; *m/z* 725.64, [SM 34∶1+Na]^+^ and *m/z* 741.59, [SM 34∶1+K]^+^; Figures 3C and 3G) as well as phosphatidylcholine signals (Figures 3D and 3H) of PC 32∶0 (*m/z* 734.66, [M+H]^+^ and *m/z* 756.60, [M+Na]^+^), PC 34∶1 (*m/z* 782.61, [M+H]^+^), PC 34∶2 (*m/z* 758.65; [M+H]^+^; *m/z* 780.62, [M+Na]^+^ and *m/z* 796.61, [M+K]^+^), PC 36∶2 (*m/z* 786.66, [M+H]^+^; *m/z* 808.67, [M+Na]^+^ and *m/z* 824.64, [M+K]^+^) and PC 36∶4 (*m/z* 804.62, [M+Na]^+^ and *m/z* 820.54, [M+K]^+^). Both lipid families appeared mostly present in mucus and membranes, as well as in the lumen at T4, showing the presence of luminal lipids during digestion. The main differences during digestion were observed by calculating and comparing peak area ratios extracted from the different images in positive ion mode (Table 2). This gives of course only qualitative information but allows us to follow the fate and distribution of either diet or endogenous lipids, throughout the digestion. As the emission yields and detection efficiency for compounds of the same family and close molecular weight can be considered equivalent [46], these ratios can bring information about the relative amount of a compound. More precisely, mass spectra and peak areas, if extracted from different histological regions of a sample, or from similar areas of different samples (in the present case from samples taken at different times), can be compared against each other by relative quantifications, provided that samples are prepared and analyzed under the same conditions [26]. Peak area ratios corresponding to MAG 16, SM 34∶1, PC 32, 34, 36, cholesterol and vitamin E decreased in comparison to T0. Conversely, the peaks area ratios increased gradually, all along digestion, for MAG 18, DAG 34, DAG 36, TAG 52 and TAG 54, all of which contain fatty acids C18 as predominant components of the sunflower oil [45]. The ratios of those containing the main C18 FA of diet oil (MAG 18, DAG 36 and TAG 54) sharply increased, while those with C18 plus C16 (DAG 34 and TAG 52) raised less.

**Table 1:** Assignment of the main neutral lipids detected *in situ*, by TOF-SIMS in positive ion mode.

assignment	Measured *m/z*	theoretical *m/z*	error (ppm)	ion
Monoacylglycerols				
MAG 16:1	311.27	311.26	32	[M+H-H_2_O]^+^
MAG 16:0	313.29	313.27	64	
MAG 18:2	337.30	337.27	89	
MAG 18:1	339.31	339.29	59	
MAG 16:0	341.34	341.31	88	
Diacylglycerols				
DAG 34:3	573.54	573.49	87	[M+H-H_2_O]^+^
DAG 34:2	575.57	575.50	122	
DAG 34:1	577.59	577.52	121	
DAG 36:4	599.57	599.50	117	
DAG 36:3	601.59	601.52	116	
DAG 36:2	603.53	603.54	−17	
Triacylglycerols				
TAG 50:3	851.82	851.71	129	[M+Na]^+^
TAG 50:2	853.82	853.73	105	
TAG 50:1	855.83	855.74	105	
TAG 52:4	877.72	877.73	−11	
TAG 52:3	879.83	879.74	102	
TAG 52:2	881.84	881.76	91	
TAG 52:1	883.85	883.77	91	

**Table 2:** Variations of the relative quantity of some peak of interest detected in positive ion mode during the digestion.

	MAG 16	MAG 18	Cholesterol	Vitamin E	DAG 34	DAG 36	SM	PC 32	PC 34	PC 36	TAG52	TAG 54
T0.5	0.85	1.39	0.96	1.03	1.49	2.63	0.87	0.92	0.83	0.84	1.48	1.50
T1	0.65	2.50	0.57	0.35	2.34	6.52	0.21	0.20	0.17	0.20	1.97	1.47
T4	0.67	2.76	0.62	0.38	2.15	6.01	0.24	0.26	0.23	0.30	2.46	3.35

Areas of peaks at T_0.5_, T_1_ and T_4_ were divided by those at T_0_.

**Figure 4:**
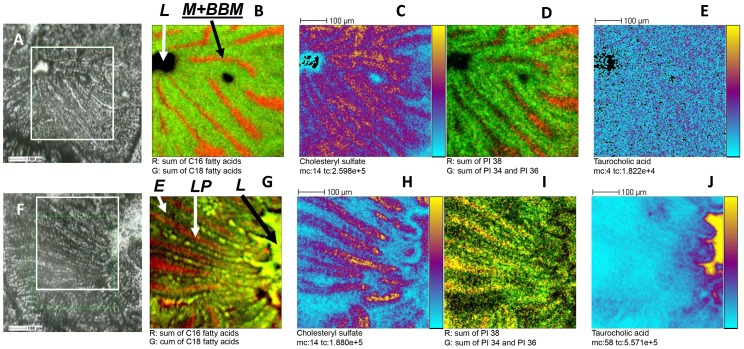
TOF-SIMS ion images recorded in negative ion mode over proximal intestine. Tissues were recovered from mice at T0 (A to E) or T4 of digestion (F to J). A and F: optical pictures, ion images were recorded in the square delimited in white. B and G: two colour overlay of the sum of C16 (*m/z* 253.21 and 255.21, in red) and C18 fatty acids (*m/z* 279.22, 281.22 and 283.24, in green). C and H: cholesterol sulfate ion images (*m/z* 465.29). D and I: two colour overlay of phosphatidylinositol containing 34 (centred at *m/z* 833.55) and 36 (centred at *m/z* 861.53) carbon atoms (green) and those containing 38 carbon atoms (centred at *m/z* 885.61) on the two fatty acids chains (red). E and J: taurocholic acid ion images (*m/z* 514.34). Primary ions Bi_3_^+^, 25 keV, fluence 5×10^11^ ions.cm^−2^, area of 500×500 µm^2^, 256×256 pixels, pixel size 2×2 µm^2^. The amplitude of the color scale corresponds to the maximum number of counts (*mc)* and could be read as [0, mc]. *tc* is the total number of counts recorded for the specified *m/z* (it is the sum of counts in all the pixels. L, duodenal lumen; M+BBM, Mucus + brush border membrane, LP, lamina propria; E, enterocytes.

In the negative ion mode, the fatty acids and phosphatidylinositol (PI) were detected but also vitamin E and cholesteryl sulfate. Images corresponding to the sum of C16 (C16:0, *m/z* 255.21; C16:1, *m/z* 253.21) and of C18 fatty acids carboxylates (C18:0, *m/z* 283.24; C18:1, *m/z* 281.22 and C18:2, *m/z* 279.22) were overlaid (Figure 4B and 4 G). Note that the detected fatty acid carboxylate ions correspond to both free fatty acids and acyl fragments from more complex lipid molecules. At T0, fatty acids containing 16 carbon atoms (in red) were essentially located in mucus, essentially at the basement of villi, and BBM, while fatty acids containing 18 carbon atoms (in green) are distributed in the whole analyzed area except the lumen. At T4 of digestion, C16 fatty acids seem to be located mostly in the lamina propria, C18 fatty acids in enterocytes while both were abundant in the lumen and mucus. The ion detected at *m/z* 465.29 and corresponding to the cholesteryl sulfate ([M-H]^−^) was distributed in the enterocytes (figures 4C and 4H), as previously reported for mouse colon [31]. The next images presented in figures 4D and 4I correspond to the sum of signals attributed to phosphatidylinositol (PI) containing 34 (centered at *m/z* 833.55, [PI 34:2-H]^−^) and 36 carbon atoms (centered at *m/z* 861.53, [PI 36:2-H]^−^) in green, and the sum of PI containing 38 carbon atoms (centered at *m/z* 885.61, [PI 38:4-H]^−^) on the two fatty acids chain in red. For the starved mouse, phosphatidylinositols 38 were principally located in the mucus, essentially at the basement of villi and in enterocyte apical and basal membranes whereas PI 34 and 36 were similarly distributed in enterocytes, lamina propria and lumen. The ion images of a peak detected at *m/z* 514.34, which was identified by MS/MS experiments using a 4800 MALDI TOF/TOF (AB Sciex, Les Ulis, France) as taurocholic acid (Figure S1), was presented in figure 4E and 4J. In proximal intestine sections of the control mouse, taurocholic acid was almost undetectable, as expected during starvation, whereas a large amount was detected in the lumen at T4 of digestion. By comparing peak area ratios from the images obtained in negative ion mode (Table 3), results obtained in positive ion mode are confirmed. Thus, ratios of C18:1 and C18:2 carboxylate ions increased still in relation to T0, while those of C18:0, C16:0 and C16:1 decreased during digestion, just as those of vitamin E, cholesteryl sulfate, PI 34, PI 36 and PI 38.The changes in fatty acid intensity were associated to a change in their distribution profiles at T4 of digestion, with a predominent localization of C16 in lamina propria, mucus and lumen, and abundance of C18 in enterocytes, mucus and lumen. Finally, a huge increase of the taurocholic acid ratio was observed, reaching 29/1 for T4/T0. A smaller increase was observed at T1, compared to T0.5 and T4, certainly due to individual variations.

**Table 3:** Variations of the relative quantity of some peak of interest detected in negative ion mode during the digestion.

	C16:1	C16:0	C18:2	C18:1	C18:0	Vitamin E	Cholesteryl sulfate	Taurocholic acid	PI34	PI36	PI38
T0.5	1.56	1.51	2.83	2.85	1.45	1.69	1.10	13.25	1.20	1.28	1.10
T1	0.75	0.80	1.73	1.95	0.90	0.67	0.78	3.18	0.76	0.86	0.73
T4	0.81	0.93	2.88	3.14	0.99	0.64	0.46	29.04	0.65	0.86	0.56

Areas of peaks at T_0.5_, T_1_ and T_4_ were divided by those at T_0_.

**Figure 5:**
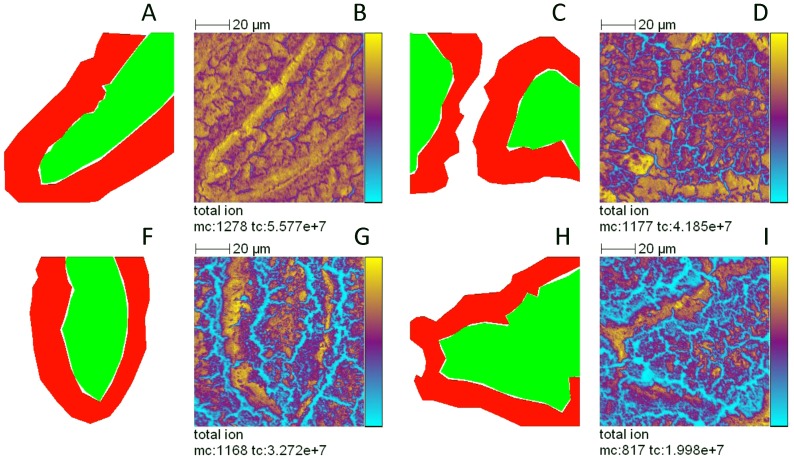
Selection of regions of interest (ROIs) for the extraction of spectrum corresponding to enterocytes and lamina propria regions. ROIs was selected from mass spectra analysis performed at T0 (A and B), T0.5 (C and D), T1 (E and F) and T4 (G and H) of digestion. A, C, E and G: regions of interest, in red, the region corresponding to enterocytes and in green, the region corresponding to lamina propria. B, D, F and H: total ion images recorded over proximal intestine of mice. Images of 100×100 µm^2^ at 256×256 pixels, pixel size 390×390 nm^2^, fluence 2×10^12^ ions.cm^−2^. The amplitude of the color scale corresponds to the maximum number of counts (*mc)* and could be read as [0, mc]. *tc* is the total number of counts recorded for the specified *m/z* (it is the sum of counts in all the pixels).

A second set of experiments was carried out on the same samples. Images of 100×100 µm^2^ area were recorded, with a pixel size of 390 nm, over proximal intestine harvested at T0, T0.5, T1 and T4 after force feeding (two acquisitions by time point). Regions of interest (ROIs) corresponding to two different image areas, i.e. lamina propria and enterocytes, were drawn and the associated mass spectra were extracted (Figure 5). As each ROI was composed of a different number of pixels (i.e. different areas), the intensities of the mass spectra were normalized towards their respective number of pixels for a proper comparison. Then, as previously, normalized areas at T0.5, T1 and T4 were divided by their relative value at T0; results are shown in Table 4. Greatest variations were observed for taurocholic acid, with relative ion peak area ratios T4/T0 close to 10 in the lamina propria region and 30 in enterocytes. The peak area ratios of the two predominant fatty acids of sunflower oil (C18: 2 and C18: 1) gradually increased again, over the digestive process, but selectively in the enterocytes and not in lamina propria. Inversely, the peak area ratios (relative to T0) concerning C18:0, C16:0 and C16:1 carboxylates remained at 1 in enterocyte regions and rather decreased in the lamina propria. A similar decrease in relative ratios was observed during digestion, for cholesteryl sulfate, SM 34:1 (also detected in the negative ion mode), phosphatidylethanolamine containing 36 carbon atoms on its two fatty acid chains, and PI 34, 36 and 38. Altogether, the TOF-SIMS analysis and the calculation of relative peak area ratios enabled to track the progressive accumulation and metabolism of the major dietary lipids, within the studied 4 hours of digestion and to selectively locate them in enterocytes.

**Table 4:** Variations of the relative quantity of the main compounds detected in negative ion mode during digestion, and for two regions.^a^

	*Lamina propria area*			*Enterocytes*		
	T0.5	T1	T4	T0.5	T1	T4
C16:1	0.92±0.00	0.55±0.05	0.36±0.04	0.98±0.50	0.95±004	0.85±0.08
C16:0	0.96±0.01	0.53±0.08	0.37±0.03	0.95±0.50	0.84±0.07	0.81±0.07
C18:2	0.96±0.10	1.09±0.30	1.06±0.09	2.11±1.04	3.08±0.73	4.19±0.36
C18:1	1.40±0.19	1.47±0.47	1.45±0.11	1.99±1.16	3.02±0.81	4.14±0.29
C18:0	0.71±0.04	0.54±0.04	0.39±0.05	1.11±0.47	1.25±0.06	1.19±0.11
Cholesteryl sulfate	0.46±0.05	0.43±0.02	0.16±0.01	0.65±0.29	0.75±0.08	0.38±0.11
Taurocholic acid	9.14±0.42	1.74±0.89	11.29±6.37	17.96±5.61	3.23±1.53	26.89±11.33
SM	0.75±0.11	0.39±0.02	0.33±0.08	0.98±0.40	0.67±0.06	0.77±0.11
PE 36	0.70±0.12	0.36±0.03	0.18±0.00	0.77±0.34	0.55±0.07	0.42±0.02
PI 34	0.66±0.12	0.36±0.04	0.14±0.01	0.76±0.28	0.56±0.08	0.33±0.04
PI 36	0.53±0.08	0.42±0.05	0.22±0.02	0.89±0.27	1.01±0.08	0.67±0.06
PI 38	0.37±0.10	0.32±0.02	0.15±0.01	0.91±0.18	0.97±0.04	0.62±0.04

**Figure 6:**
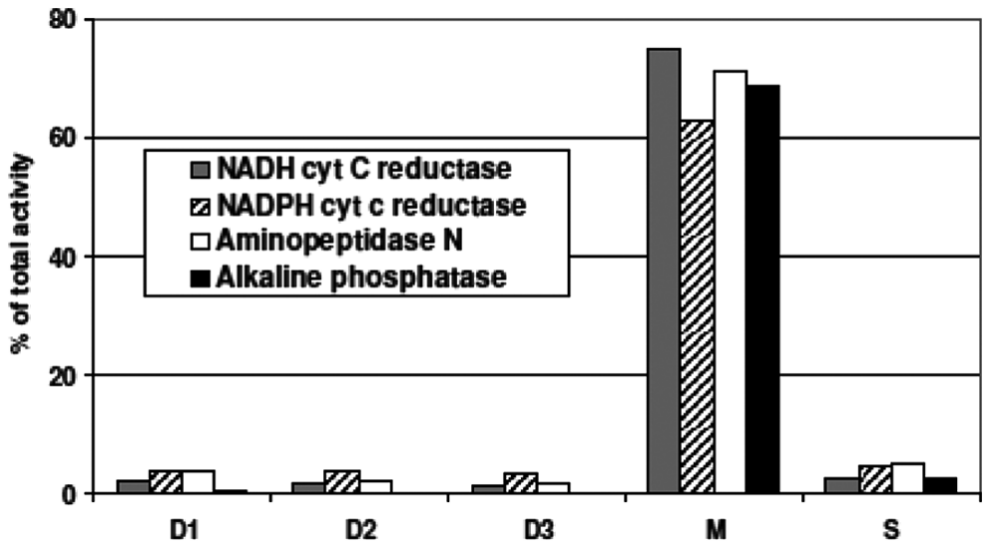
Enzymatic detection of membrane markers in LD. In order to appreciate the purity of the isolated LD, the activities of four intestinal membrane proteins were detected, which are respective markers of the Golgi apparatus (NADH cytochrome C reductase), the ER (NADPH cytochrome C reductase) and the apical brush border of enterocytes (aminopeptidase N and alkaline phosphatase). Activities were measured in I, D1, D2 and D3, in M and the final infranatant S, calculated by U/mg of proteins and expressed as % of total activity in the intestinal homogenate (I). A typical experiment representative of two is presented.

Values were calculated in two separate intestinal regions and are expressed in means ± SD. Normalized areas of peaks at T0.5, T1 and T4 were divided by those at T0.

a According to schemes in Figures 6A, 6C, 6F and 6H.

### Isolation and characterization of intestinal lipid droplets

Lipid digestion was associated to the formation of different kind of lipid containing droplets in intestine. Enterocytes contain cytosolic lipid droplets and microsomal pre- or formed chylomicrons whereas lamina propria has secreted chylomicrons or iHDL (Figure 1), [17], [47], [48], [49], [50]. In order to characterize these lipid structures, we developed an isolation procedure permitting to purify three populations of droplets by sequential ultracentifugations. Among them, D1 and D3 have respective sizes and densities similar as those of chylomicrons and HDL, while that of D2 was intermediate. Additionally the chylomicrons engaged in the secretory pathway, were separated within microsomes in the sample M.

The LD purity was assessed by measuring enzymatic activities of membrane markers: aminopeptidase N and alkaline phosphatase from apical BBM, NADH and NADPH cytochrome C reductase from, respectively, the endoplasmic reticulum and the Golgi apparatus. As expected, the four enzyme activities were high in M that contained plasma membranes and microsomes (Figure 6). Conversely, very low levels are detected in the droplets D1, D2 and D3 and the remaining cytosol S. They are therefore considered free of contaminating membranes, confirming their purity. Cytosolic contamination of D1, D2 and D3 was also avoided by meticulous removal of the top cushions.

### Lipidomic analysis

**Figure 7:**
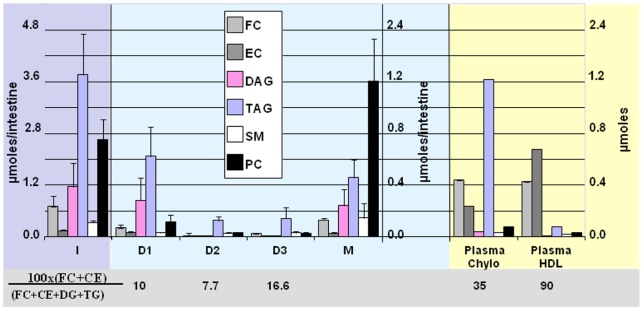
Comparative lipid analysis in intestinal lipid droplets and microsomes/membranes in the intestine after 4 h of fat digestion. The repartition of free (FC) or esterified (EC) cholesterol, diacylglycerol (DAG), triacylglycerol (TAG), sphingomyelin (SM) and PC in the intestinal homogenate I, the CLD D1, D2 and D3, the membranes + microsomes (M) and plasma chylomicrons and HDL. The data are presented in A) as µmoles/intestine corresponding to the mucosa from the 10 first cm of proximal intestine, with distinct scales for either I (highlighted in gray) or D1,D2,D3 and M (in blue) and in µmoles for lipoproteins (yellow).

Neutral lipids, sphingomyelins, ceramides and PCs were quantified in I, D1, D2, D3 and M and expressed as µmoles/intestine, i.e. mucosa from 10 cm of proximal intestine containing about 15 mg of proteins (Figure 7) and in plasma chylomicrons and HDL. In order to compare the distribution of lipids, these results were also shown as percentages of the total contents in the homogenate I (Table 5). Interestingly, D1, D2 and D3 contained TAG, FC and CE, being the major lipids of CLD [22], [51] or chylomicrons (see figure 7) [51], but also DAG. The results in Figure 7 also allow us to compare the intestinal lipid composition D1 and D3 with mouse plasma lipoproteins isolated by similar centrifugations chylomicrons and HDL, respectively. They clearly have distinct compositions with at first, an absence of DAG in the lipoproteins and very different ratios of total cholesterol/total neutral lipids. Cholesterol was indeed much more abundant in lipoproteins than CLD. As expected from the isolation procedure, fatty acids (TAG, DAG) were mainly recovered in D1 attesting for their large and fat-rich structure and in M containing microsomal chylomicrons. Cholesterol was also abundant in D1 representing 9.65+2.83% of total FC and about 20% of total EC but also in M which included FC from membranes and chylomicrons (Table 5). As expected, the droplets D2 and D3 contained much less FA. D3 was relatively rich in cholesterol over D1 and D2, which is predominantly esterified. As compared to I or M, PC was much less abundant in D1, D2 and D3 with respective contents of 2.22±0.57, 0.91±0.32 and 1.44±0.56 µmoles/mg of proteins which are close to values found in LD isolated from adipocytes or fibroblasts [52], [53]. The droplets also contained significant amounts of SM and ceramides representing up to 10% of the total intestinal contents.

**Table 5:** Repartition of each lipid between D1, D2, D3 and M as expressed in percentage of its respective content in I.

	D1	D2	D3	M
		% of total amounts in the homogenate I		
FC	9.65±2.83	1.18±0.43	3.02±1.59	22.32±9.11
EC	20.40±6.19	1.98±0.62	7.63±3.81	21.10±7.67
DG	25.89±9.97	3.12±1.57	4.10±3.02	14.09±6.95
TG	17.05±7.31	3.22±1.24	2.57±1.13	29.44±9.99
SM	9.33±1.52	5.48±2.22	10.91±5.32	71.35±13.01
Cer	8.38±1.84	5.69±2.16	4.72±3.21	ND
PC	5,71±1.80	1.26±0.37	1.14±0.51	58.73±21.31

The same data as in figure 7 are presented in means of % ± SEM from n = 5 separate experiments concerning D1, D2, D3 and M, and n = 2 for chylomicrons and HDL.

### Detection of LD proteins and lipid metabolism enzymes

**Figure 8:**
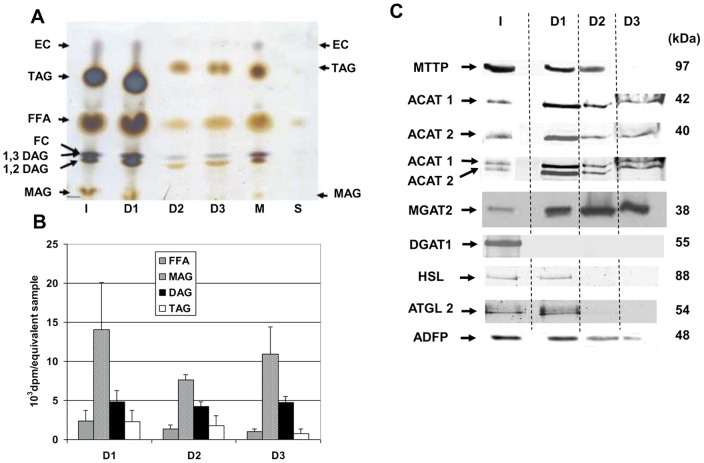
Lipidomic analysis. Intestinal LD contains TAG but also DAG, MAG and FFA and lipid metabolizing enzymes A) TLC analysis of neutrals lipids from initial homogenate (I), the LD D1, D2 and D3, the fraction M containing microsomes and membrane and the last infranatant (S). Lipids were stained using 25% H_2_SO_4_ and heated what led to stain in blue free (FC) and esterified (EC) cholesterols and in brown free fatty acids (FFA), monoacylglycerol (MAG), diacylglycerol (DAG, either 1,2 DAG or 1,3 DAG) and triacylglycerol (TAG). Sample volumes were adapted to permit concomitant lipid visualization in a unique thin layer. S was almost exempt of lipids even after concentration. B) Acyl esterase assay were performed with equivalent samples of D1, D2 and D3. Lipids were extracted, separated by TLC, stained with iodine vapor. The bands corresponding to FFA, MAG, DAG and TAG were scrapped and the incorporated radio-activity was quantified by liquid scintillation counting. Data are expressed in means of 10^3^ dpm/equivalent sample ± SEM, n = 3 separate experiments. C) Immunodetection of ADFP (adipocyte differenciation-related protein), MTTP (microsomal triglyceride transfer protein), ACAT1 and ACAT2 (acetyl-CoA cholesterol acyl transferase), MGAT2 (monoacyl glycerol acyl transferase 2), HSL (Hormone sensitive lipase) and ATGL (adipose triglyceride lipase). ACAT1 and ACAT2 were selectively detected in distinct bands that are clearly distinguished after co-incubation with anti-ACAT1 and anti-ACAT2 antibodies. For each sample, equivalent amounts of proteins (60°µg) were subjected to SDS-PAGE and transferred on nitrocellulose membrane. All data in the figure are representative of 3 separate experiments.

In view of the detection of DAGs in intestinal droplets, we have also analyzed them in TLC, which separates FFA, MAG, DAG and TAG. This confirmed the presence DAG and TAG but also significant amounts of FFA, and MAG (Figure 8A). We cannot exclude that digestive or intestinal lipases have hydrolyzed triacylglycerol, during homogenization and purification, despite the use of 20 µM of orlistat to inhibit them. However, the fatty acid ratios remained unchanged using more of the inhibitor suggesting that LD may contain diet FA not yet fully integrated in TAG. We therefore wondered if they could hold some transferases, esterases or lipases explaining the observed diversity in neutral lipid.

Indeed, as shown in Figure 8B, an acyl-transferase activity was detected leading to significant incorporation of radiolabelled oleoyl-CoA in DAG or TAG of D1, D2 and D3, which was in a range of 1/10 of that found in M. Because DGAT1 and MGAT2 were shown to be the major acyl-transferases involved in TAG synthesis during digestion, we studied their expression by Western-blots and identified MGAT2 in D1, D2 and D3 but not DGAT1 (Figure 8C). Interestingly, a significant amount of radioactivity was incorporated in the FFA and MAG isolated by TLC, suggesting that a lipase activity has hydrolyzed newly synthetized DAG or TAG. Among those expressed in enterocytes, we detected HSL (hormone sensitive lipase) and ATGL (adipose triglyceride lipase) in D1. Interestingly, ACAT1 and ACAT2 were additionally identified in D1, D2 and D3 and MTTP in D1 and D2 D2 but not D3. The anti-ACAT1 and anti-ACAT2 antibodies detected unique bands of, respectively, 42 and 40 apparent kDa with very similar patterns. However, co-hybridization with both antibodies showed no cross reactivities between the two antibodies. Unfortunately, we were unsuccessful to detect any ACAT activity using the method described by Chang et al. [54], even in I and M, probably because of an enzyme inhibition by excess fatty acids or EC. In accordance to previous studies [19], [23], adipophilin or ADFP (adipocyte differentiation-related protein), a characteristic constituent of CLD is mainly identified in D1 but also in D2 and D3.

## Discussion

It is well recognized that the digestion of fats by enterocytes is associated with the formation of LD and lipoproteins for transient storage, metabolism, and transport of lipids to the circulation. This study provides a detailed characterization of intestinal lipids during their digestion in vivo in mouse, after an acute high fat feeding. Imaging, biochemical and mass spectrometry analysis reveals several features of the intestinal fatty acids and cholesterol metabolism. TOF-SIMS analysis permits a precise localization of lipids in the duodenum of starved and fed mice, representing footprints of in vivo events. It also allows the extraction of each mass spectrum in certain areas of interest, such as the enterocyte monolayer, and the comparison of relative lipid contents at different times of digestion, from the number of detected pixels. The biochemical study, in turn, led to the isolation of intestinal lipid droplets and quantitative analysis of lipids, together with the identification of several metabolic activities and the corresponding enzymes.

First, as previously reported, the histological staining of neutral lipids by ORO reveals that numerous LD have invaded intestinal enterocytes, the largest being visible by light microscopy. Secreted chylomicrons are also observed in the lamina propria and lymphatic vessels. TOF-SIMS provides a unique way to detect various duodenal lipids and track their distribution during digestion. Before their intestinal absorption, diet TAG and EC are hydrolyzed by pancreatic enzymes and emulsified in mixed micelles with other lipids by bile salts. Mass spectrometry imaging of mouse duodenum clearly shows this process, detecting taurocholic acid, FA but also PC and SM filling the lumen during digestion but absent at T0. They very probably represent these micelles, even if a partial release of lipids could also occur by artifact, during tissue cutting. Another interesting feature of mass spectrometry imaging is to follow the uptake and movement of major fatty acid triglycerides of sunflower oil, which are linoleic acid (C18: 2, 60%) and oleic acid (C18: 1, 20%). They are identified either free or associated to glycerol in MAG 18, but also in the metabolites DAG 36, DAG 34 and TAG 54 or 52.

Lipid localization in mucus and membranes is also revealed for PC, PI 38, SM and C16 FA as well as glycerol associated fatty acids, which clearly underlines the apical side of enterocytes along the villi, but also, to a lower extent, the basal side. By contrast, PI 34 and PI 36 and C18 FA are present in both enterocytes and lamina propria at either T0 or T4 of digestion.

The analysis was refined, with images recorded with a pixel size of 390 nm over an area of 100×100 µm^2^. Analyses were then performed by selectively extracting mass spectra from two regions of interest: enterocytes and lamina propria. The comparison of peaks area ratios, at the times T0.5, T1 and T4 after oil gavage, *versu*s those at T0, permits to follow lipid distribution during their digestion. Extracting ROI clearly shows a significant and progressive increase until T4, in the ratios of C18: 2 and C18: 1 in the enterocytes but not lamina propria. This is consistent with the known duration of lipid digestion with an accumulation of absorbed FA in enterocytes that lasts for several hours, leading to lipid storage, metabolism and chylomicron synthesis [55]. So, even after 4 hours of digestion, the absorbed lipids in intestine are mainly located in enterocytes but not in the lamina propria, reflecting the fast elimination of secreted lipoproteins in the circulation. This is in agreement with a previous identification of TAG in the cytosol of enterocytes by in vivo coherent anti-strokes Raman scattering imaging, during fat digestion in mouse [19]. The fatty acids C16 and C18:0, representing, respectively, only 7% and 5% of the total sunflower oil FA, shows a slight increase in the whole intestine at T0.5 (Figure 4I), which is no longer visible at T1 and T4. By contrast, the cholesterol ion intensity, during mass spectroscopy imaging, is not visibly modified over the digestive process, despite its presence in the gavage oil. This could be explained by a strong endogenous expression of this sterol in the intestine, masking any increase during digestion.

As expected, taurocholic acid is detected during the whole process of digestion but not at T0, confirming a delivery of bile in the intestinal lumen. It is also detected in enterocytes and lamina propria, reflecting its known intestinal re-absorption, which is major in ileum but also occurs in the duodenum, in the process of enterohepatic circulation and homeostasis [56]. Intestinal contents of vitamin E is increased at T0.5 of digestion, as compared to T0, but decreased in T1 or T4, probably because it is also secreted in chylomicrons and HDL, as previously shown [57]. Interestingly, a decrease is observed in the intestinal relative ratios of many endogenous phospholipids: PC, PI, PE, SM, cholesteryl sulfate and fatty acids C16, which progress all along digestion. This could be partially explained by a decrease of endogenous lipid synthesis during the digestion process, but also by a transfer of SM, ceramides and phospholipids, in D1, D2, D3, and then in lipoproteins. This study highlights the potentialities offered by the TOF-SIMS technique, its sensitivity, its precision and its capability to simultaneously follow the fate of several lipid compounds, which allows tracking biological events within the criteria of location, temporality and metabolism.

**Figure 9:**
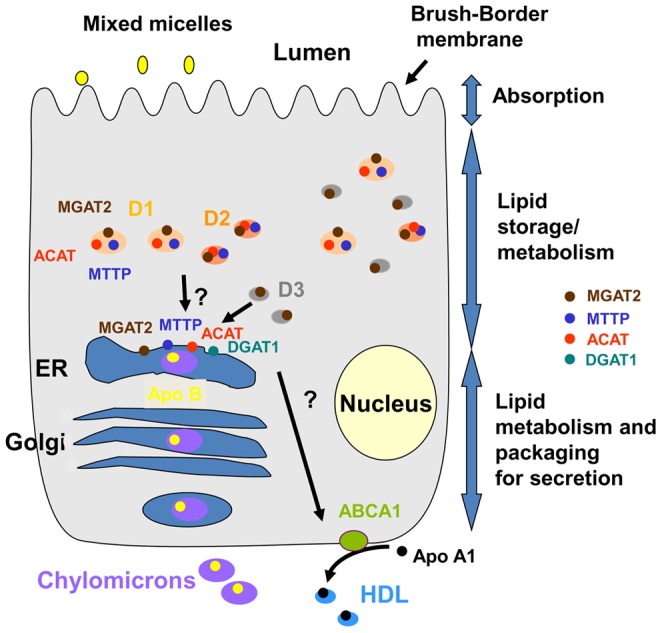
Schematic representation of the digestion of lipids in enterocytes. The absorption, traffic and metabolism of lipids were identified, leading to the secretion of chylomicrons by the secretory apparatus or HDL. D1, D2, D3 and ER were localized as well as the enzymes responsible for the esterification of FA (DGAT1 and MGAT2) or cholesterol (ACAT1 and 2), MTTP and ABCA1. The apolipoproteins ApoA1 and ApoB were also identified, which are characteristic of, respectively, HDL, and chylomicrons.

During digestion, hydrolyzed FA and cholesterol are absorbed by the apical membranes of enterocytes and are intracellularly transported to the endoplasmic reticulum, by an unknown mechanism [4] where they are further metabolized and transferred to chylomicrons. However, as much as 30% of digested cholesterol is directed to a second pathway, largely unknown inside the enterocytes but conducting to a basal secretion of iHDL [4], [17]. In addition to HDL and chylomicrons, cytosolic lipid droplets are found in abundance in enterocytes and are supposed to be involved in lipid storage, pending ER mobilization for chylomicron synthesis [58], [59]. The intestinal lipid droplets remain poorly characterized except several recent studies [19], [20], [23]. With the aim of characterizing, we originally purify three populations of intestinal LD plus microsomal chylomicrons, separately recovered in M. Because of the isolation procedure and of the high preponderance of fatty acids in the enterocytes and not the lamina propria (Table 4, [18]), we can estimate that most of D1, D2 and D3 originated from the cytosol of enterocytes. This is further consistent with the difference we observed by lipidomic analysis between D1, D2, D3 and plasma chylomicrons and HDL. At last, ADFP, a marker of CLD in various cells, is clearly identified by Western-blots, in D1, D2 and D3. From the isolation procedure, the D1 droplets are supposed to be large and rich in FA as chylomicrons, the D2 smaller and less rich and D3, even less. This was confirmed by the lipidomic analysis with high levels of DAG or TAG in D1 corresponding to 19%±2 of total intestinal FA, which appeared equivalent to the 17%±7 in M, containing chylomicrons and membranes. FA contents are much lower in D2 and D3 by only corresponding 2 to 3% of total content. Interestingly, D3 contained as much as 3%±1.6 of the total intestinal FC and 7.6%±3.8 of EC whereas D1 hold 9.6%±2.8 of FC and 20.4%±6.2 of EC. This is in agreement with previous results showing that about 30% of the absorbed cholesterol is addressed to iHDL, during physiological fat digestion in mouse [17]. Thus the cholesterol repartition between D1 and D3 is matching that of secreted lipoproteins. One of these populations could be responsible for the movement of lipids in the way of the intestinal production of HDL. This pathway being independent from MTTP, the D3 droplets constitute a good candidate which do not contain this protein and have low levels in FA, although this remains to be characterized. We summarize in figure 9 our results and the potential contributions of cytosolic LD in fat digestion.

Adapted from the methodology allowing plasma lipoprotein purification, our isolation procedure provides three distinct populations of intestinal CLD having unique lipid and protein contents, with only the largest D1 probably detectable by optical microscopy. The LD also appear to be pure from contaminating membranes, as judged from the quasi-absence of enzymatic activities of membrane or microsomes markers and characteristic low content of some lipids such as PC and SM, as compared to M. They are also clearly distinct from chylomicrons and HDL which much lower proportion of cholesterol (FC+EC) as compare to lipoproteins or other CLD [52] but also significant amounts of FA which should be intermediates in TAG synthesis. They thus appear as transient structures of incompletely metabolized lipids being quite distinct from general composition of CLD in other cells [22], [52], [53]. In the ER, absorbed lipids are known to be further metabolized in TAG by monoacylglycerol acyltransferase-2 (MGAT-2) and diacylglycerol acyltransferase-1 (DGAT-1) but also MGAT3 expressed in humans but not in mouse, while acylCoA cholesterol acyl transferases (ACAT) enzymes, especially ACAT2, re-esterify FC in EC [15], [41], [60], [61], [62], [63],. Because of the characteristic compositions of D1, D2 and D3, we studied their metabolic propensity to metabolize neutral lipids. Indeed, previous studies identified in CLD some enzymes of lipid biosynthesis and hydrolysis in several cell types, including the human differentiated Caco-2 enterocytes supplied with mixed micelles [23], [64]. For instance, two enzymes involved in triglyceride synthesis have been identified in CLD as DGAT1 in macrophages [65] and DGAT2 in adipocytes [66] which are conversely absent from Caco-2 CLD [23]. In all our three intestinal droplet populations, DGAT1 was not detected (not shown) but MGAT2 was clearly identified by Western-blot, as well as by its corresponding enzymatic activity, leading to DAG and TAG synthesis. The radioactivity incorporated into the MAG and FFA of D1, D2, D3 may in turn arise from degradation by lipases of the labeled DAG and TAG. For that matter, we identified the cytosolic proteins HSL and ATGL in D1 droplets which have been similarly found in Caco2 CLD [23]. ACAT 1 and ACAT2 were also detected in D1, D2 and D3 where it could act in cholesterol esterification, while HSL was previously shown to hydrolyze cholesterol ester [67]. MTTP was also detected in intestinal D1 and D2 in accordance with its detection in Caco-2 CLD, by proteomic [23]. It is well known that MGAT2, MTTP and ACAT1 and 2 are mainly expressed in the ER. The LD/I expression ratios of MGAT, MTTP and ACAT1 per µg of proteins are between 1 to 3 but only of 0.02 for the reticulum marker NADPH cytochrome C reductase. This huge difference strongly suggests that these four transferases are truly expressed in intestinal CLD rather than coming from ER contaminations. This shows that CLD in enterocytes may vary in size and composition in proteins, lipids and in metabolic capacities.

## Conclusion

This study shows a precise analysis by TOF-SIMS mass spectrometry imaging of lipid repartition in mice intestine, before and during fat digestion, as well as the isolation to purity and biochemical and lipidomic characterization of several digestive CLD. Our results show that cytosolic lipid droplets contain some enzymes able to synthesis or hydrolyze TAG and EC, which is of major importance in enterocytes. These results underline the essential involvement of chylomicrons in FA absorption and the dual function of chylomicrons and iHDL in cholesterol absorption. The challenge of this field will now be to clarify how intestinal physiology could influence lipid metabolism in order to lead to new pharmaceutical targets to complete existing lipid-lowering strategies.

## Supporting Information

Figure S1 Mass spectrum analysis of taurocholic acid. A: Structure of taurocholic acid. B and C: MS/MS spectra (1 keV, CID on, precursor ion: *m/z* 514.28) in negative ion mode recorded with a MALDI TOF/TOF spectrometer over a proximal intestine section of mouse sacrificed after 4 hours of digestion (B) and on a spot of a standard solution of taurocholic acid at 1 mg.mL^−1^ in water:methanol 1∶1 (v/v) (C). A solution of α–cyano-4-hydroxycinnamic at 10 mg.mL-1 (water:acetonitrile 1:1 v/v) was used as MALDI matrix.
(TIF)
