# Supplementary figures and images for: Lipidomic and Spatio-Temporal Imaging of Fat by Mass Spectrometry in Mice Duodenum during Lipid Digestion
Source: PLoS One. 2013 Apr 3;8(4):e58224. doi: 10.1371/journal.pone.0058224 (PMC3616127; doi:10.1371/journal.pone.0058224)

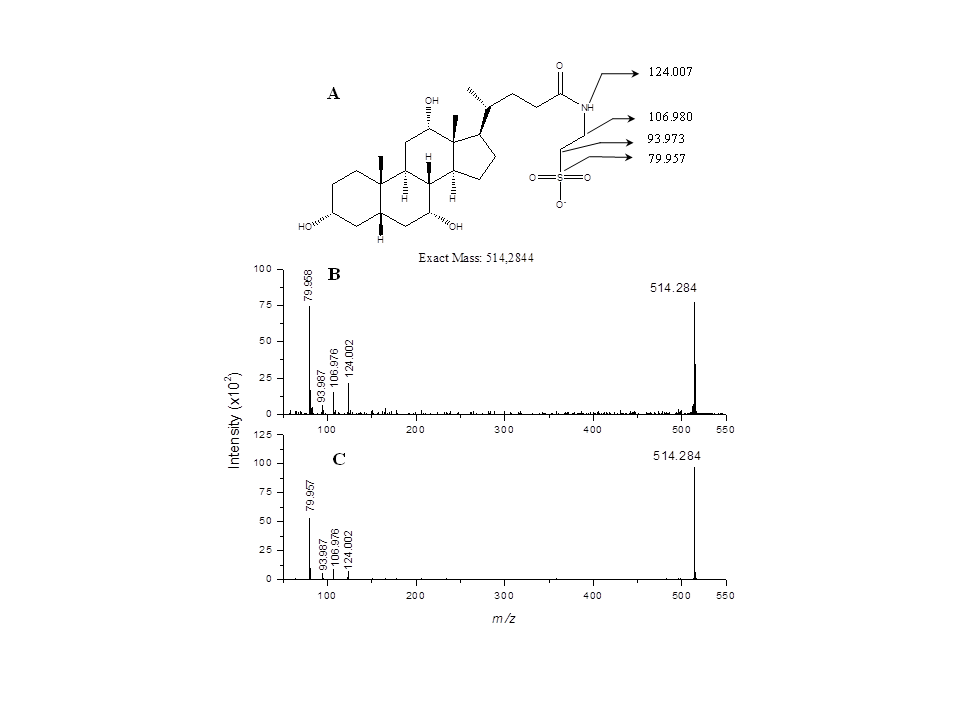

Supplement: Figure S1 — Mass spectrum analysis of taurocholic acid. A: Structure of taurocholic acid. B and C: MS/MS spectra (1 keV, CID on, precursor ion: m/z 514.28) in negative ion mode recorded with a MALDI TOF/TOF spectrometer over a proximal intestine section of mouse sacrificed after 4 hours of digestion (B) and on a spot of a standard solution of taurocholic acid at 1 mg.mL−1 in water:methanol 1∶1 (v/v) (C). A solution of α–cyano-4-hydroxycinnamic at 10 mg.mL-1 (water:acetonitrile 1:1 v/v) was used as MALDI matrix. (TIF) [file pone.0058224.s001.tif]
